# Plasma membrane perforation by GSDME during apoptosis-driven secondary necrosis

**DOI:** 10.1007/s00018-021-04078-0

**Published:** 2021-12-31

**Authors:** Elke De Schutter, Jana Ramon, Benjamin Pfeuty, Caroline De Tender, Stephan Stremersch, Koen Raemdonck, Ken Op de Beeck, Wim Declercq, Franck B. Riquet, Kevin Braeckmans, Peter Vandenabeele

**Affiliations:** 1 VIB Center for Inflammation Research, 9052 Ghent, Belgium; 2 Department of Biomedical Molecular Biology, Ghent University, 9052 Ghent, Belgium; 3 Center of Medical Genetics, University of Antwerp and Antwerp University Hospital, 2650 Antwerp, Belgium; 4 Laboratory of General Biochemistry and Physical Pharmacy, Faculty of Pharmaceutical Sciences, Ghent University, 9000 Ghent, Belgium; 5 Université de Lille, CNRS, UMR 8523-PhLAM-Physique des Lasers Atomes et Molécules,, 59000 Lille, France; 6 Department of Applied Mathematics, Computer Science and Statistics, Ghent University, 9000 Ghent, Belgium; 7 Plant Sciences Unit, Flanders Research Institute for Agriculture, Fisheries and Food, 9820 Merelbeke, Belgium; 8 Center for Oncological Research, University of Antwerp and Antwerp University Hospital, 2610 Antwerp, Belgium; 9 Université de Lille, 59000 Lille, France

**Keywords:** Gasdermins, Cell death, Membrane permeabilization, Influx, Efflux, Dextrans

## Abstract

Secondary necrosis has long been perceived as an uncontrolled process resulting in total lysis of the apoptotic cell. Recently, it was shown that progression of apoptosis to secondary necrosis is regulated by Gasdermin E (GSDME), which requires activation by caspase-3. Although the contribution of GSDME in this context has been attributed to its pore-forming capacity, little is known about the kinetics and size characteristics of this. Here we report on the membrane permeabilizing features of GSDME by monitoring the influx and efflux of dextrans of different sizes into/from anti-Fas-treated L929sAhFas cells undergoing apoptosis-driven secondary necrosis. We found that GSDME accelerates cell lysis measured by SYTOX Blue staining but does not affect the exposure of phosphatidylserine on the plasma membrane. Furthermore, loss of GSDME expression clearly hampered the influx of fluorescently labeled dextrans while the efflux happened independently of the presence or absence of GSDME expression. Importantly, both in- and efflux of dextrans were dependent on their molecular weight. Altogether, our results demonstrate that GSDME regulates the passage of compounds together with other plasma membrane destabilizing subroutines.

**Supplementary Information:**

The online version contains supplementary material available at 10.1007/s00018-021-04078-0.

## Introduction

Apoptosis, the best-known form of regulated cell death, is essentially a containment and recycling program that prepares the cell corpse for efficient phagocytosis [1]. However, when phagocytes are absent or the phagocytic capacity is insufficient, apoptotic cells progress to necrotic plasma membrane permeabilization called apoptosis-driven secondary necrosis, which results in a more inflammatory environment [2–5]. The gasdermin (GSDM) protein family gained a lot of interest as plasma membrane permeabilizers during regulated cell death [6–8]. Gasdermin D (GSDMD) is proteolytically activated by caspase-1 and -4 leading to inflammasome-mediated pyroptosis [9, 10] and the GSDMD-dependent release of pro-inflammatory cytokines such as interleukin-1β [11]. Similarly, apoptosis-driven secondary necrosis is driven by the activation of gasdermin E (GSDME) [12, 13]. To entail its effect, caspase-3-mediated cleavage induces the release of GSDME’s cytotoxic N-terminal p30 fragment from the auto-inhibiting C-terminal domain, which is followed by plasma membrane recruitment and plasma membrane permeabilization [12, 14, 15]. Nevertheless, GSDME may not be the only mechanism responsible for secondary necrosis. GSDME expression is dispensable for secondary necrosis following NLRC4-mediated apoptosis in macrophages [16] or UV irradiation-induced apoptosis in human T cells and monocytes [17].

After cleavage, GSDM proteins were shown to oligomerize and bind to plasma membrane phospholipids [14, 18], suggesting that GSDMs establish plasma membrane permeabilization by perforation. Moreover, the structure of GSDMD and mouse GSDMA3 revealed more mechanistic insights in how the N-terminal domain is able to form pores [19, 20]. Using cryo-electron microscopy, it was discovered that the N-terminal domains of GSDMA3 form a large, 27-fold β-barrel-stave protein pore with an inner diameter of 18 nm [19]. In addition, 26- and 28-fold oligomerization structures were reported with similar dimensions as the dominant 27-subunit GSDMA3 pore [19]. In contrast, GSDMD assemblies were reported to have a 31- to 34-fold symmetry [20], suggesting significant variability in oligomerization among different GSDM proteins. In addition, the N-terminal domain of GSDMD assembles into dynamic arc- and slit-shaped oligomers before they finally transform to stable ring-shaped oligomers with varying diameters ranging from 13.5 till 33.5 nm [21, 22].

Unlike GSDMA3 and GSDMD, the characteristics of GSDME pore formation are currently unknown. Therefore, to gain insight into the membrane permeabilizing behavior of GSDME and its role in apoptosis-driven secondary necrosis, we applied two in vitro approaches. With the assumption that GSDME forms pores in the plasma membrane, influx or efflux of macromolecules, such as fluorescently labeled dextrans, is expected to happen when cells are exposed to apoptotic stimuli. Monitoring the uptake of fluorescently labeled dextrans in apoptotic cells is quite straightforward, only requiring the addition of the dextrans to the culture medium after apoptosis induction. However, monitoring efflux is less obvious as the cells should be pre-loaded with the dextrans in a manner that does not interfere with cellular processes such as proliferation or without inducing apoptosis by itself. Therefore, we selected nanoparticle-sensitized photoporation, which is an emerging intracellular delivery technique that enables direct cytosolic delivery of membrane-impermeable macromolecules in virtually every cell type with minimal impact on the cellular homeostasis [23–29]. This technique makes use of photothermal nanoparticles, such as gold nanoparticles (AuNPs), which are incubated with cells and bind to the plasma membrane. Upon irradiation by a short, yet intense laser pulse, the AuNPs become heated, resulting in the evaporation of the surrounding water and the formation of quickly expanding water vapor nanobubbles (VNBs) around the AuNPs. The mechanical forces resulting from the expansion and collapse of those VNBs lead to the generation of localized pores in the plasma membrane [27, 30]. Through those transient plasma membrane pores, which are repaired within seconds to minutes, an exchange of molecules between the intra- and extracellular compartment can happen [25, 26, 28, 29]. Importantly, under controlled conditions, complete cellular recovery is reported within 24 h upon laser treatment with minimal effect on the cellular homeostasis [23, 24, 26].

Here, we studied the membrane permeabilizing behavior of GSDME during apoptosis-driven secondary necrosis and attempted to elucidate whether this process is characterized by discrete pore sizes and/or whether GSDME pores grow over time. To this end, we developed GSDME-deficient L929sAhFas cells carrying a doxycycline-inducible system for GSDME expression allowing the exploration of secondary necrosis in the absence or presence of GSDME in the same cellular context. We reveal that the absence of GSDME delays nuclear staining by SYTOX Blue (SB), as cells remain longer in the sublytic phase, while phosphatidylserine (PS) exposure was not affected. Next, we investigated the involvement of GSDME in the influx and efflux of fluorescently labeled dextrans of different sizes during apoptosis-driven secondary necrosis induced by anti-Fas. We provide evidence that pore formation during apoptosis-driven secondary necrosis is a gradual process that already supports a GSDME-dependent influx of fluorescently labeled dextrans before the nuclear DNA of dying cells is stained by SB. Furthermore, the influx method allowed us to make an estimation of molecular sizes able to pass the GSDME pore. In contrast, efflux of fluorescently labeled dextrans seemed to occur independently of GSDME combined with the fact that only significant dextran loss was observed when cells were already stained by SB.

## Materials and methods

### Cell culture

L929sAhFas cells and derivates were cultured in Dulbecco’s Modified Eagle Medium supplemented with 10% of Fetal Bovine Serum (v/v), l-glutamine (2 mM) and sodium pyruvate (400 mM). Cells were cultured in 37 °C in a humidified atmosphere containing 5% CO_2_ and were regularly tested against mycoplasma contamination.

### Generation of gasdermin E-deficient L929sAhFas cells

Single guide RNAs (sgRNA) targeting the exon 4 of *Gsdme* were selected using the Wellcome Trust Sanger Institute Genome Editing database (WGE) [31] and were manufactured by Thermo Fischer Scientific. The sgRNA sequences are listed in Supplementary Table S1. The sgRNA oligo sequence was cloned in BpiI-digested pSpCas9(BB)-2A-GFP carrying *Streptococcus pyogenes* WT Cas9 (Addgene, plasmid no. 48138). The sgRNA Cas9 plasmid was transfected into L929sAhFas cells via jetPRIME transfection reagent (Polyplus-transfection). 4 µg plasmid was added per 25 000 cells and incubated for 4 h at 37 °C, 5% CO_2_ after which the culture medium was replaced and cells were further incubated for 4 days at 37 °C, 5% CO_2_. Next, cells were harvested and GFP-positive cells were sorted using a FACSAria III (BD Biosciences). Effective genomic interruption of *Gsdme* was confirmed with PCR and Sanger sequencing. Allele editing was analyzed using TIDE [32]. The PCR and sequencing primers used are listed in Supplementary Table S1.

### Generation of a stable gasdermin E-inducible L929sAhFas cell line

The L929sAhFas iGSDME cell line was obtained by transduction of *Gsdme* KOcl2 L929sAhFas cells with a pDG2-mGSDME-blast plasmid. This is a tetracycline-inducible derivative of pLenti6 (Life Technologies) containing a blasticidin selection marker [33], in which the coding sequence of murine GSDME was cloned. Upon lentiviral transduction, the stably transduced cells were selected with 5–10 µg/ml blasticidin.

### Analysis of phosphatidylserine exposure and cell death kinetics

L929sAhFas iGSDME cells were seeded in 24-well suspension plates (100 × 10^3^ cells/well) in the presence or absence of doxycycline (Sigma-Aldrich, 1 µg/ml) and stimulated the next day with 125 ng/ml anti-Fas (clone CH11, Upstate). Cell death parameters were analyzed after incubation between 0 h and 10 h of anti-Fas treatment. 1 h before measurement, fluorescent probes were added to the culture medium: 1.25 µM of SYTOX Blue nucleic acid stain and 7.5 nM of Annexin V Alexa Fluor 488 conjugate (Molecular Probes). Subsequently, samples were measured by flow cytometry using a four-laser BD Fortessa or three-laser BD LSR II (BD Biosciences) and data were analyzed using FlowJo 10.7.1.

### Western blotting

L929sAhFas iGSDME cells were pretreated with 1 µg/ml doxycycline (Sigma-Aldrich) to allow GSDME expression. L929sAhFas and L929sAhFas iGSDME cells were incubated for 8 h with 250 ng/ml anti-Fas (clone CH11, Upstate) at 37 °C, 5% CO_2_, after which they were harvested and washed twice in ice-cold phosphate-buffered saline (PBS). Next, cells were lysed using ice-cold RIPA lysis buffer (50 mM Tris–HCl; pH 7.5; 150 mM NaCl; 1 mM EDTA; 0.5% sodium deoxycholate; 1% Triton X-100 and 0.1% SDS) freshly supplemented with EDTA-free complete protease inhibitor cocktail tablets and phosphatase inhibitor cocktail tablets (Roche Diagnostics Belgium N.V.). Extracted proteins were separated on 12% sodium dodecyl sulfate (SDS) polyacrylamide gels and transferred onto nitrocellulose membranes (Amersham Bioscience). Membranes were blocked using Tris-buffered saline containing 0.05% Tween® 20 (TBS-T) and 5% non-fat dry milk (Biorad) followed by incubation with anti-GSDME (ab215191, abcam) or anti-actin (69100, MP Biomedicals) antibodies. After incubation with the horseradish peroxidase (HRP)-linked donkey anti-rabbit IgG or HRP-linked sheep anti-mouse (Amersham Biosciences), blots were revealed using a Western Lightning Plus-ECL (PerkinElmer).

### Intracellular delivery of FITC-labeled dextrans by nanoparticle-sensitized photoporation

AuNPs with a core size of 60 nm were in-house synthetized using the Turkevich method and coated with the cationic polymer poly(diallyldimethylammonium chloride) (PDDAC) as reported before [34].

To determine the AuNP concentration that provides optimal photoporation results, L929sAhFas (130 × 10^3^) were seeded in 24-well plates and allowed to attach overnight at 37 °C, 5% CO_2_. Next, cells were incubated for 30 min (37 °C, 5% CO_2_) with different concentrations of AuNPs (2, 4, 6, 8 and 16 × 10^7^ AuNPs/mL), washed with PBS to remove unbound AuNPs, and replenished with fresh culture medium containing 5 mg/ml FITC-labeled dextrans (Sigma-Aldrich) of 10 kDa (FD10). Subsequently, cells were photoporated using an in-house built laser irradiation set-up equipped with a nanosecond pulsed laser (5 ns pulse duration, *λ* = 532 nm, Tor, Cobolt) and a galvano scanner (Thorlabs, THORLABS-GVS002.SLDPRT) for rapid beam scanning across the samples. A fixed laser pulse fluence (optical energy per unit area) of 0.86 J/cm^2^ was applied. After laser treatment, FD10-diluted medium was removed and cells were washed twice with culture medium and once with PBS followed by cell detachment using 0.25% trypsin–EDTA. At last, cells were measured for their FD10 content by flow cytometry using a three-laser BD LSR II (BD biosciences) and data were analyzed using FlowJo 10.7.1.

For loading with FITC-labeled dextrans in the function of efflux experiments, L929sAhFas iGSDME cells (650 × 10^3^ cells/well) were seeded in 6-well plates and allowed to attach overnight at 37 °C, 5% CO_2_. The same protocol as described before was used. In this case, cells were incubated with the optimal AuNP concentration (6 × 10^7^ AuNPs/ml) for 30 min. After washing away of unbound AuNPs, culture medium was added containing FITC-labeled dextrans (Sigma-Aldrich) of 4 kDa (FD4), 10 kDa (FD10), 40 kDa (FD40), 70 kDa (FD70), 150 kDa (FD150), 250 kDa (FD250), 500 kDa (FD500) or 2000 kDa (FD2000). For all sizes, a concentration of 5 mg/ml was used, except for 2000 kDa for which the concentration was increased to 10 mg/ml. Cells were subsequently photoporated using a fixed laser pulse fluence of 0.86 J/cm^2^ after which the dextran-containing medium was removed and cells were washed twice with culture medium. After 2 h of incubation (37 °C and 5% CO_2_), the same procedure was repeated a second time to further increase the percentage of fluorescently labeled cells. Finally, cells were detached using 0.25% trypsin–EDTA and re-seeded at 100 × 10^3^ cells/well in 24-well suspension plates in the presence or absence of doxycycline (Sigma-Aldrich, 1 µg/ml) and allowed to grow overnight (37 °C, 5% CO_2_).

### Influx and efflux of fluorescently labeled dextrans and cell death analysis

For influx experiments, L929sAhFas iGSDME cells were seeded in 24-well suspension plates (100 × 10^3^ cells/well) in the presence or absence of doxycycline (Sigma-Aldrich, 1 µg/ml) and treated the next day with 250 ng/ml anti-Fas (clone CH11, Upstate) for 10 h every 2 h. Afterwards, cells were harvested by gently pipetting up and down and were immediately centrifuged at 400 g for 5 min at 4 °C. After removing the supernatant, the cells were resuspended in culture medium containing 0.5 mg/ml Texas Red-labeled dextrans (Invitrogen and Nanocs) of 10 kDa (TR10), 40 kDa (TR40), 70 kDa (TR70) or 2000 kDa (TR2000) and incubated for 5 min at room temperature. Next, cells were centrifuged again for 5 min at 400 g and 4 °C, washed and resuspended in culture medium containing 2.5 µM SYTOX Blue (Molecular Probes) for nuclear staining. Samples were subsequently measured by flow cytometry using a four-laser BD Fortessa (BD Biosciences) and data were analyzed using FlowJo 10.7.1.

For efflux experiments, L929sAhFas iGSDME cells were preloaded with FITC-labeled dextrans (Sigma-Aldrich) using nanoparticle-sensitized photoporation and re-seeded in 24-well suspension plates in the presence or absence of doxycycline (Sigma-Aldrich, 1 µg/ml). One day after photoporation, re-seeded L929sAhFas iGSDME were treated with 250 ng/ml anti-Fas (clone CH11, Upstate) for 10 h every 2 h. Subsequently, cells were stained with SYTOX blue (Molecular probes) at a concentration of 2.5 µM after which they were collected by gently pipetting up and down and measured by flow cytometry using a three-laser BD LSR II (BD Biosciences). Data were analyzed using FlowJo 10.7.1.

### CellTiter-Glo® cell viability assay

In view of determining the optimal AuNP concentration, cell viability was assessed 2 h after laser treatment using the CellTiter-Glo® luminescent cell viability assay (Promega) following the manufacturer’s protocol. Briefly, the culture medium was replaced by equal amounts of fresh culture medium and CellTiter-Glo® reagent and cells were mixed for 30 min using an orbital shaker at 120 rpm. After allowing stabilization of the luminescent signal for 15 min, equal volumes of each well were transferred to an opaque well plate and luminescence was recorded by a GloMax™ Luminometer (Promega).

### Statistical analysis

Results are presented as means ± SD. Statistical analysis of PS exposure and SB staining as a function of time were performed using PRISM 8 software (GraphPad) using a two-way ANOVA with the Geisser-Greenhouse correction (matched values were stacked into a subcolumn). For the influx and efflux dataset, homogeneity of variances and data normality were checked graphically (boxplots, QQPlots, respectively). Analysis of the influx of Texas Red-labeled dextrans was done making use of a generalized linear model (GLM), Poisson family. To study the effect of dextran size on either the SB-negative (SB-) and SB-positive (SB+) cells, the factor variables doxycycline addition and measured point in time (0, 2, 4, 6, 8 and 10 h of anti-Fas treatment) and the discrete variable dextran size were included in the model. To study the effect of doxycycline on the influx of Texas Red-labeled dextrans, doxycycline addition, the measured point in time (0, 2, 4, 6, 8 and 10 h of anti-Fas treatment) and dextran size were all set as factor variables in the GLM. Multiple comparisons were made making use of the package “multcomp” [35].

To analyze the efflux of FITC-labeled dextrans, the dataset was split into three populations (SB−, SB low+, SB high+). For both datasets, a GLM (Gaussian family) was fitted to study the effect of dextran size and doxycycline on the efflux of dextrans. The factor variables doxycycline, measured point in time (0, 2, 4, 6, 8 and 10 h of anti-Fas treatment) and the discrete variable dextran size were included in the model. Again, multiple comparisons were made using the package “multcomp”. All analyses were done in R, version 4.3 on three biological replicates of each data-set [36].

Differences with a *p*-value < 0.05 were considered significant and indicated as followed: ns = not significant; *p < 0.05; **p < 0.01; ***p < 0.001.

## Results

### Gasdermin E accelerates plasma membrane permeabilization during apoptosis-driven secondary necrosis as measured by SYTOX Blue-mediated nuclear staining.

**Fig. 1:**
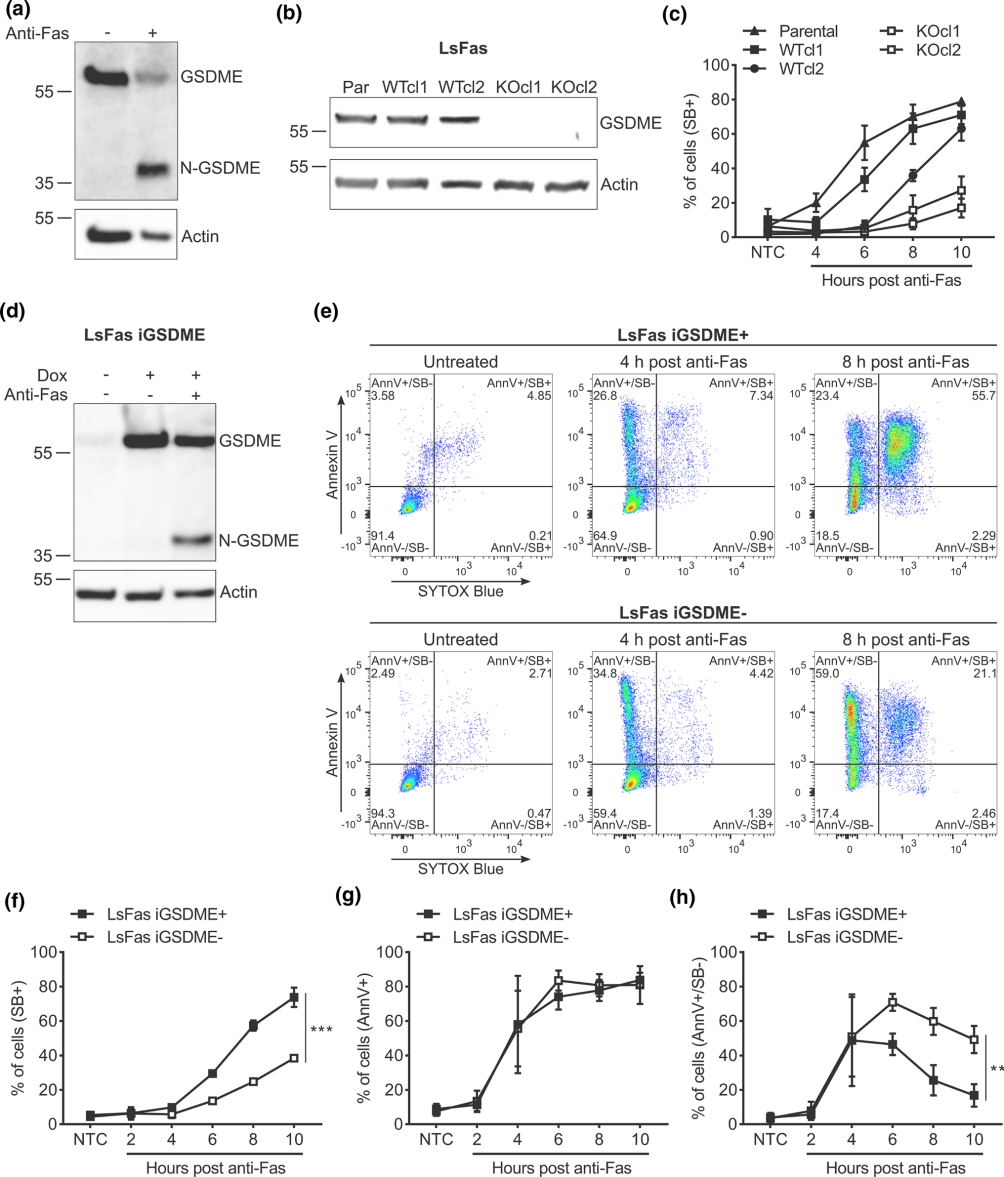
Impact of GSDME expression on apoptosis-driven secondary necrosis in L929sAhFas cells. **a** Expression and proteolytic cleavage of GSDME in L929sAhFas upon anti-Fas treatment. **b** Expression of GSDME in different L929sAhFas clones upon CRISPR/Cas 9 gene editing. **c** Cell death kinetics of parental, *Gsdme* WT and KO L929sAhFas clones measured by SB staining via flow cytometry. **d** Expression of GSDME in L929sAhFas iGSDME cells upon doxycycline treatment. Subsequent treatment with agonistic anti-Fas antibodies promotes the generation of the active 35 kDa N-terminal fragment (N-GSDME). **e–h** Flow cytometry analysis of L929sAhFas iGSDME cells with (L929sAhFas iGSDME+) or without (L929sAhFas iGSDME−) doxycycline-induced GSDME expression during apoptosis-driven secondary necrosis. **e** Representative flow cytometry dot plots after 4 h or 8 h treatment with anti-Fas. **f** Levels of secondary necrotic (SB+) cells, **g** cells exposing PS (AnnV+) and **h** PS single-positive (AnnV+ /SB−) cells in L929sAhFas iGSDME cells treated with anti-Fas. *AnnV* Annexin V; *Dox* doxycycline; *GSDME*, gasdermin E; *KO* knockout; *LsFas* L929sAhFas; *NTC* non-treatment control; *Par* parental; *SB* SYTOX Blue; *WT* wild-type

As conflicting findings were reported on the contribution of GSDME to apoptosis-driven secondary necrosis [12, 13, 16, 17], we decided to investigate the GSDME function in the murine fibrosarcoma cell line L929 stably expressing the human Fas receptor (L929sAhFas). Treatment of L929sAhFas cells with agonistic anti-human Fas antibodies induces apoptosis and caspase-3 activation via the FADD/caspase-8-dependent proteolytic pathway [2, 37–40]. As expected, treating L929sAhFas with anti-Fas resulted in the generation of a GSDME fragment of ~ 35 kDa (Fig. 1a), indicating proteolytic activation of GSDME by caspase-3 as previously reported [12, 41]. To investigate the role of GSDME in anti-Fas-mediated apoptosis, we generated *Gsdme* knockout (KO) L929sAhFas clones by CRISPR/Cas9 gene editing (Fig. 1b). Next, we investigated whether the loss of GSDME expression in *Gsdme* KO L929sAhFas clones (KOcl1 and KOcl2) affected the kinetics of the uptake of the cell-impermeable DNA-binding fluorescent dye SB during apoptosis-driven secondary necrosis (Fig. 1c). Upon anti-Fas treatment, *Gsdme* KO L929sAhFas clones (KOcl1 and KOcl2) showed a delay in the uptake of SB compared to the parental cells and *Gsdme* wild-type (WT) clones in which CRISPR/Cas9 gene-editing failed to interrupt *Gsdme* (WTcl1 and WTcl2, Fig. 1c), indicating delayed plasma membrane permeabilization in absence of GSDME expression, as concluded from the SB staining. To confirm that this delay was GSDME dependent and did not result from a clonal effect, *Gsdme* KOcl2 L929sAhFas was reconstituted with a doxycycline-inducible mGSDME construct using viral transduction, hereafter referred to as L929sAhFas iGSDME cells. Treatment of these cells with doxycycline resulted in the expression of GSDME that was cleaved to its active form upon treatment with anti-Fas (Fig. 1d). To compare the progression of apoptosis between GSDME-expressing (L929sAhFas iGSDME+) and GSDME-deficient (L929sAhFas iGSDME−) cells in more detail, we measured nuclear staining by SB and membrane surface PS exposure with Annexin V (AnnV) (Fig. 1e). Reconstitution of GSDME expression in L929sAhFas iGSDME cells by doxycycline treatment accelerated SB-positivity upon anti-Fas treatment compared to GSDME-deficient cells (Fig. 1e, f), suggesting that the plasma membrane permeabilization kinetics are slower in cells lacking GSDME. Interestingly, upon anti-Fas treatment, both L929sAhFas iGSDME+ and iGSDME- cells displayed a similar increase in membrane surface PS exposure, as measured by AnnV staining (AnnV+ cells, Fig. 1g), and total apoptotic and secondary necrotic cell death combined (AnnV+ /SB− and SB+ cells, Fig. S1). Given this similar kinetics of AnnV-positivity, the slower SB-positivity in L929sAhFas iGSDME- cells correlates with a prolonged PS single-positive stage (AnnV+ /SB−, Fig. 1h). The number of AnnV+ /SB− cells starts to decline in L929sAhFas iGSDME+ conditions, 4 h after anti-Fas treatment, while in cells lacking GSDME a prolonged PS single-positive stage (AnnV+/SB−) can be observed (Fig. 1h). These data suggest that the initial progression of apoptotic signaling, leading to PS exposure, is not affected by GSDME expression, but GSDME is required to speed up plasma membrane permeabilization as measured by SB staining. Moreover, our results indicate that the loss of GSDME expression delays but does not prevent plasma membrane permeabilization thereby suggesting that other, GSDME-independent, plasma membrane permeabilization mechanisms exist during apoptosis-driven secondary necrosis in L929sAhFas cells.

### Gasdermin E pore formation supports influx of 10 kDa dextrans independently of plasma membrane permeabilization kinetics

**Fig. 2:**
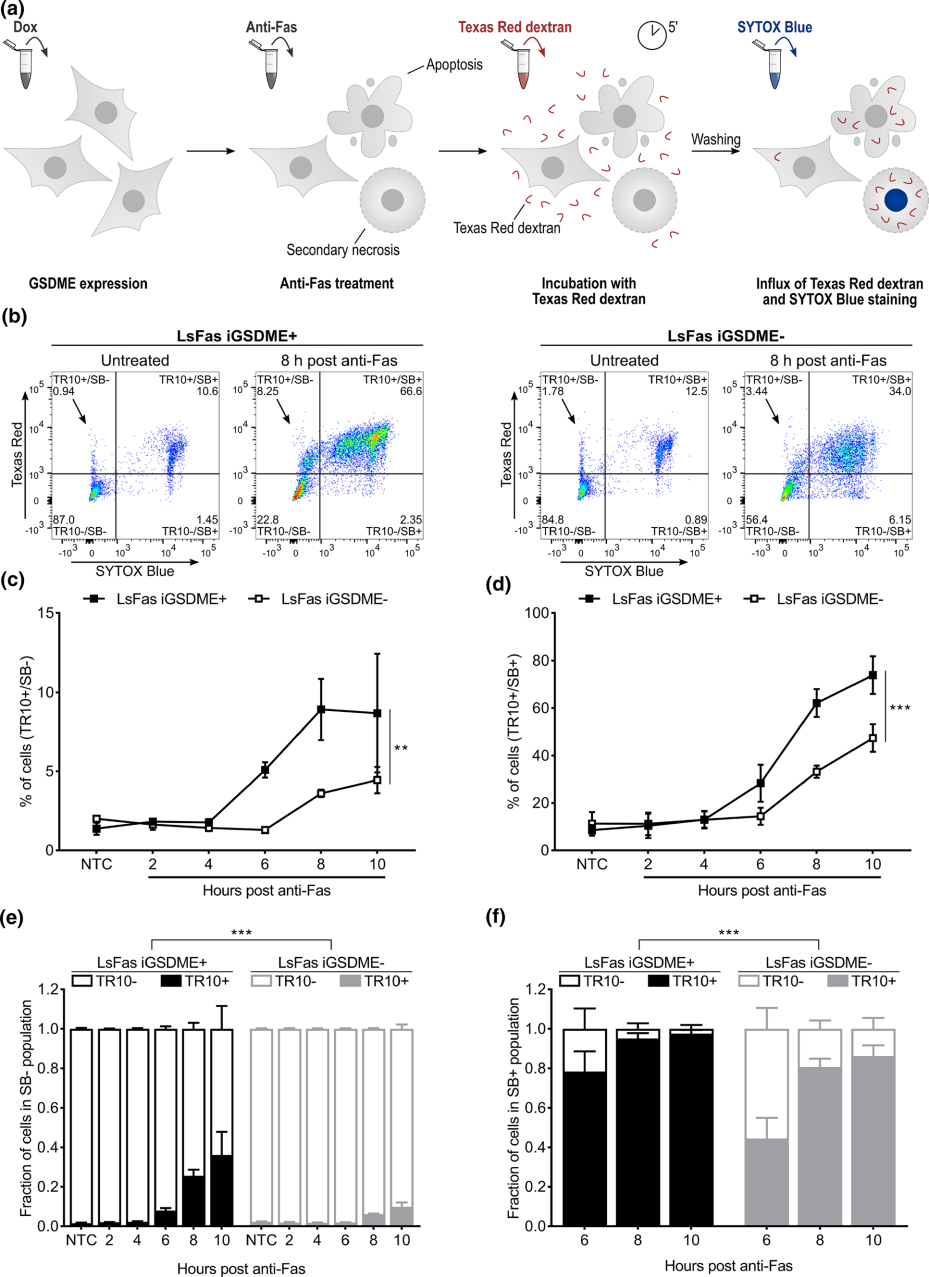
Monitoring of Texas Red-labeled dextran 10 kDa (TR10) influx in L929sAhFas iGSDME cells during apoptosis-driven secondary necrosis. **a** Principle of Texas Red-labeled dextran staining of L929sAhFas iGSDME cells. **b–f** Flow cytometry analysis of L929sAhFas iGSDME cells with (L929sAhFas iGSDME+) and without (L929sAhFas iGSDME-) doxycycline-induced GSDME expression during apoptosis-driven secondary necrosis. **b** Representative plots of L929sAhFas iGSDME cells untreated and after treatment with anti-Fas for 8 h. **c** Levels of Texas Red single-positive cells (TR10+/SB−) in L929sAhFas iGSDME cells upon anti-Fas treatment. **d** Levels of Texas Red and SB double-positive (TR10+/SB+) cells in L929sAhFas iGSDME cells upon anti-Fas treatment. **e** Fraction of Texas Red positive (TR10+) and Texas Red negative (TR10−) cells in the SB− population. **f** Fraction of Texas Red positive (TR10+) and Texas Red negative (TR10−) cells in SB+ population. *Dox* doxycycline; *GSDME* gasdermin E; *LsFas* L929sAhFas; *SB* SYTOX Blue; *NTC* non-treatment control; *TR* Texas Red

Having confirmed that GSDME expression accelerates nuclear DNA staining by SB (Fig. 1f), we next evaluated whether GSDME-dependent membrane permeabilization supports the influx of large, membrane-impermeable macromolecules as well. Texas Red-labeled dextrans with a size of 10 kDa (TR10) were used to this end, allowing convenient quantification of influx by flow cytometry (Fig. 2a). Texas Red signal was assessed in SB− and SB+ cells separately, according to the zones indicated in Fig. 2b. Remarkably, treatment of L929sAhFas iGSDME cells with anti-Fas followed by incubation with TR10 and SB resulted in a TR10 single-positive population (Fig. 2b, TR10+ /SB−, black arrow) and a double-positive population (Fig. 2b, TR10+/SB+), suggesting that TR10 can already enter the cells before SB stains the nuclear DNA (Fig. 2b). Unlike Fas-induced PS exposure, which happened independently of GSDME expression (Fig. 1g), TR10 tends to accumulate in twice as much SB- L929sAhFas iGSDME+ cells compared to SB- cells lacking GSDME (Fig. 2c). As the apoptotic phase and thus apoptotic blebbing and cellular fragmentation (SB- status) of L929sAhFas GSDME+ cells is limited compared to cells lacking GSDME (Fig. 1f), the TR+/SB− events seen in the flow cytometry data are probably not resulting from nucleus-free cellular fragments. Instead, this observation suggests that the influx of TR10 is enhanced by GSDME-dependent plasma membrane permeabilization in the sublytic phase, before staining by SB. Noteworthy, the TR+/SB− population seems relatively minor. As measurements were done every 2 h and GSDME-expressing cells proceed rapidly from the TR+/SB− to the TR+/SB+ stage (Fig. 1f), we hypothesize that the time-interval between both stages is shorter than 2 h and thus explains the rather low percentage of cells that resides in the TR10+/SB− single-positive stage. However, we cannot rule out the possibility that only a minor subset of cells are stained by TR10 before SB staining while most cells are stained simultaneously with TR10 and SB. Consistent with the SB- population, the TR10+/SB+ population was higher in GSDME-expressing cells (Fig. 2d), which is expected as we showed that, upon anti-Fas treatment, staining by SB was accelerated in L929sAhFas iGSDME+ cells (Fig. 1f). Interestingly, we also observed a minor TR10−/SB+ population (Fig. 2b), pointing toward cells that become lytic with pores too small to allow the influx of TR10. It is conceivable that these cells died due to the massive presence of small pores, although this is just speculation and our experiment does not provide proof for this.

Next, we investigated whether the GSDME-related difference observed in TR10 influx is simply the result of delayed cell death kinetics, measured by SB staining, in L929sAhFas iGSDME- or a direct consequence of the absence of the GSDME pore itself. To neutralize the difference in cell death kinetics in our results, as pointed out in the previous section (Fig. 1f), we assessed TR10 uptake in the SB− and SB+ population by normalizing the number of TR10− and TR10+ cells against the total number of cells in the respective populations (Fig. 2e, f). Apparently, the fraction of TR10 + cells upon anti-Fas treatment in SB- L929sAhFas iGSDME− cells was limited and significantly less compared to when GSDME was present (Fig. 2e). This suggests that GSDME pore formation itself allows the influx of TR10 before SB-mediated nuclear staining. In SB+ cells, TR10 entered L929sAhFas iGSDME- cells much more easily, pointing to other permeabilization mechanisms taking place as well during apoptosis-driven secondary necrosis (Fig. 2f). Still, TR10 entered L929sAhFas iGSDME+ cells significantly more, indicating that plasma membrane permeabilization by GSDME enhances TR10 influx. Interestingly, both in SB− (Fig. 2e) and SB+ cells (Fig. 2f), the fraction of TR10+ cells increased over time. This suggests that the longer cells remain SB- upon anti-Fas treatment, the more cells get permeabilized, thereby promoting the entrance of TR10 while cells are in the sublytic phase and are not yet stained by SB. It also conceivable that more cells are permeabilized with the additional formation of larger pores allowing the entrance of TR10, as the molecular size sieving is determined by its largest pore. Though, we do not have hard experimental evidence for such consecutive small and large pore formation. Altogether, our results indicate that the presence of GSDME promotes a faster and increased influx of TR10 in both SB− and SB+ cells during apoptosis-driven secondary necrosis.

### Gasdermin E pore formation facilitates the influx of large dextrans in a size-dependent manner

**Fig. 3:**
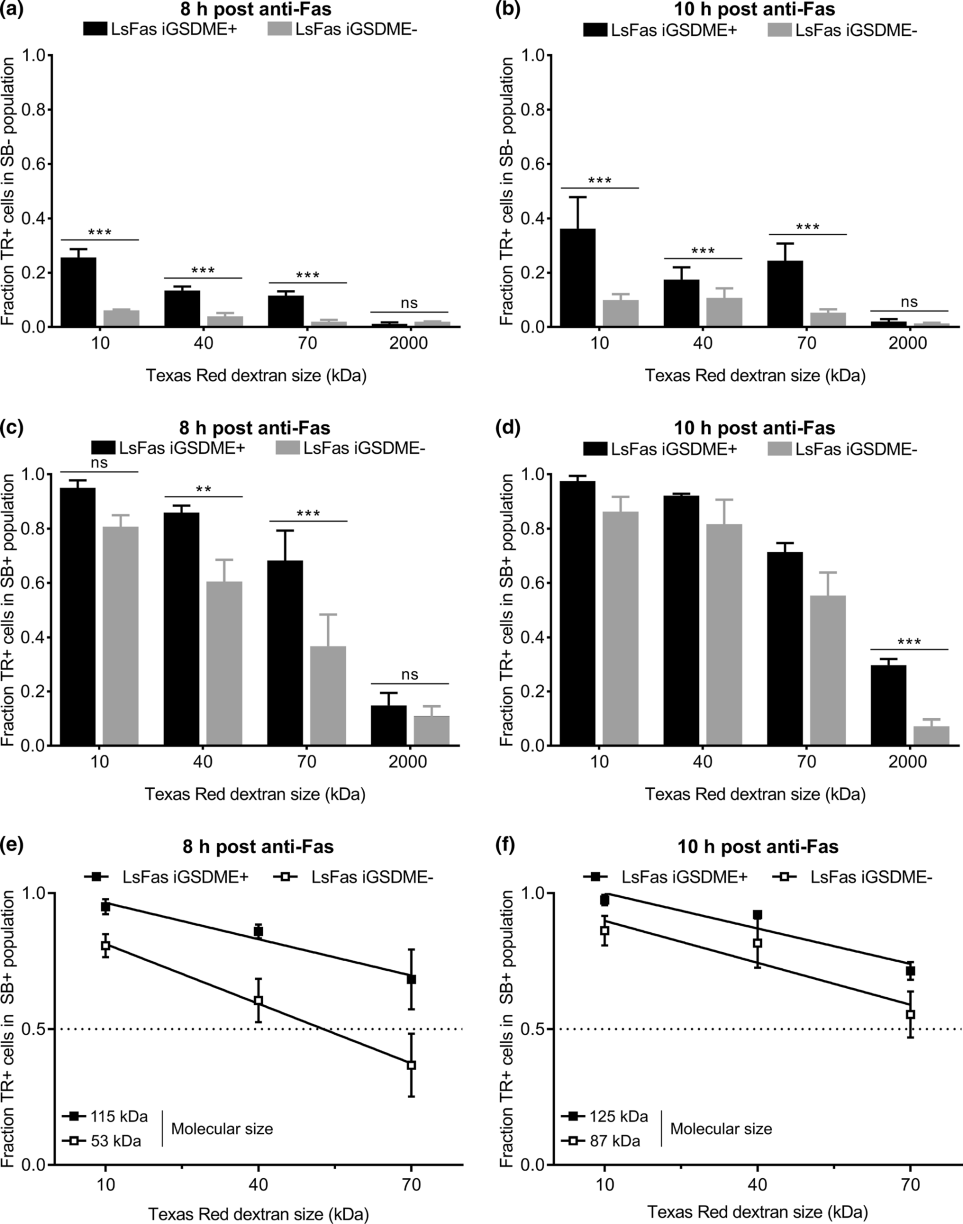
Influx of Texas Red-labeled dextrans of 10 kDa (TR10), 40 kDa (TR40), 70 kDa (TR70) and 2000 kDa (TR2000) in L929sAhFas iGSDME during apoptosis-driven secondary necrosis. **a–f** Flow cytometry analysis of Texas Red-labeled dextran uptake in L929sAhFas iGSDME cells with (L929sAhFas iGSDME+) and without (L929sAhFas iGSDME−) doxycycline-induced GSDME expression after 8 h and 10 h treatment with anti-Fas. **a**,** b** Fraction of the SB- population that is positive for various sizes of Texas Red-labeled dextrans. **c**,** d** Fraction of the SB+ population that is positive for various sizes of Texas Red-labeled dextrans. **e**,** f** Linear fit of data points for the fractions of the SB+ population positive for TR10, TR40 and TR70. Intersection of this line with the dotted line provides a rough estimation of molecular sizes that can enter 50% of the SB+ population. *LsFas* L929sAhFas; *SB* SYTOX Blue; *TR* Texas Red

As TR10 is able to enter L929sAhFas iGSDME cells even when GSDME is absent, we wondered whether there is a molecular weight above which dextrans can no longer enter GSDME-deficient cells. Therefore, we examined the influx of Texas Red-labeled dextrans of 40 kDa (TR40), 70 kDa (TR70) and of 2000 kDa (TR2000) and how this is affected by GSDME expression in L929sAhFas iGSDME cells upon anti-Fas treatment. Influx of Texas Red-labeled dextrans was again assessed in both SB− and SB+ cells separately. Overall, upon 8 h (Fig. 3a) and 10 h (Fig. 3b) of treatment with anti-Fas, absence of GSDME expression significantly reduced the influx of all dextran sizes in SB- L929sAhFas iGSDME cells, while influx clearly did happen when GSDME was present, except for TR2000. Although prolonged anti-Fas treatment promoted the influx of Texas Red-labeled dextrans up to 70 kDa in both L929sAhFas iGSDME+ and iGSDME− cells, this was still significantly lower in absence of GSDME (Fig. 3b). Moreover, the uptake of Texas Red-labeled dextrans in SB- cells decreased with increasing dextran size, both in the absence (10 h, *p* < 0.01) and presence (8 h, *p* < 0.05; 10 h, *p* < 0.01) of GSDME expression (Fig. 3a, b). These observations point toward pore formation during apoptosis-driven secondary necrosis with a rather variable instead of a fixed size. These results suggest that GSDME pores, formed in SB- L929sAhFas iGSDME cells, allow the passage of dextrans up to at least 70 kDa while they exclude the entrance of Texas Red-labeled dextrans equal or larger than 2000 kDa. Importantly, note that the GSDME-dependent influx of Texas Red-labeled dextrans happened prior to SB staining, suggesting that GSDME membrane permeabilization during apoptosis-driven secondary necrosis does not occur concurrently with nuclear DNA staining by small SB molecules (0.4 kDa) and already happens prior to secondary necrosis.

The GSDME dependency for the influx in SB+ L929sAhFas iGSDME cells was less pronounced for TR10 after treatment with anti-Fas for 8 h (Fig. 3c), whereas after 10 h influx of sizes 10–70 kDa revealed to be not significant between L929sAhFas iGSDME+ and iGSDME− cells (Fig. 3d). Nevertheless, GSDME expression clearly promoted the entrance of TR2000 in SB+ L929sAhFas iGSDME+ cells after treatment with anti-Fas for 10 h (Fig. 3d). Although this suggests that GSDME pores in SB+ cells might even favor the entrance of molecules up to 2000 kDa, most of the cells (~ 70%) were still negative for TR2000. Furthermore, similar to the influx in SB- cells, influx of Texas Red-labeled dextrans significantly decreased with increasing dextran size. On average, dextran size had an overall statistical significant effect on the influx 8 h after anti-Fas treatment in L929sAhFas iGSDME- cells (Fig. 3c, *p* < 0.001) and 10 h after anti-Fas treatment in L929sAhFas iGSDME+ (Fig. 3d, *p *< 0.05) and iGSDME− (Fig. 3d, *p* < 0.001) cells. Linear fit of the data points for TR10, TR40 and TR70 upon 8 h (Fig. 3e) and 10 h (Fig. 3f) of anti-Fas treatment, allowed us to interpolate the size of molecules that can enter 50% of the cells (Fig. 3e, f). According to our calculations, GSDME expression would allow the uptake of molecules between 115 (8 h) and 125 kDa (10 h) in 50% of the SB + L929sAhFas iGSDME + cells, while the absence of GSDME limits the molecular size to 53 (8 h) and 87 kDa (10 h).

### Nanoparticle-sensitized photoporation constitutes a suitable method for introducing dextrans into cells without influencing apoptosis kinetics

**Fig. 4:**
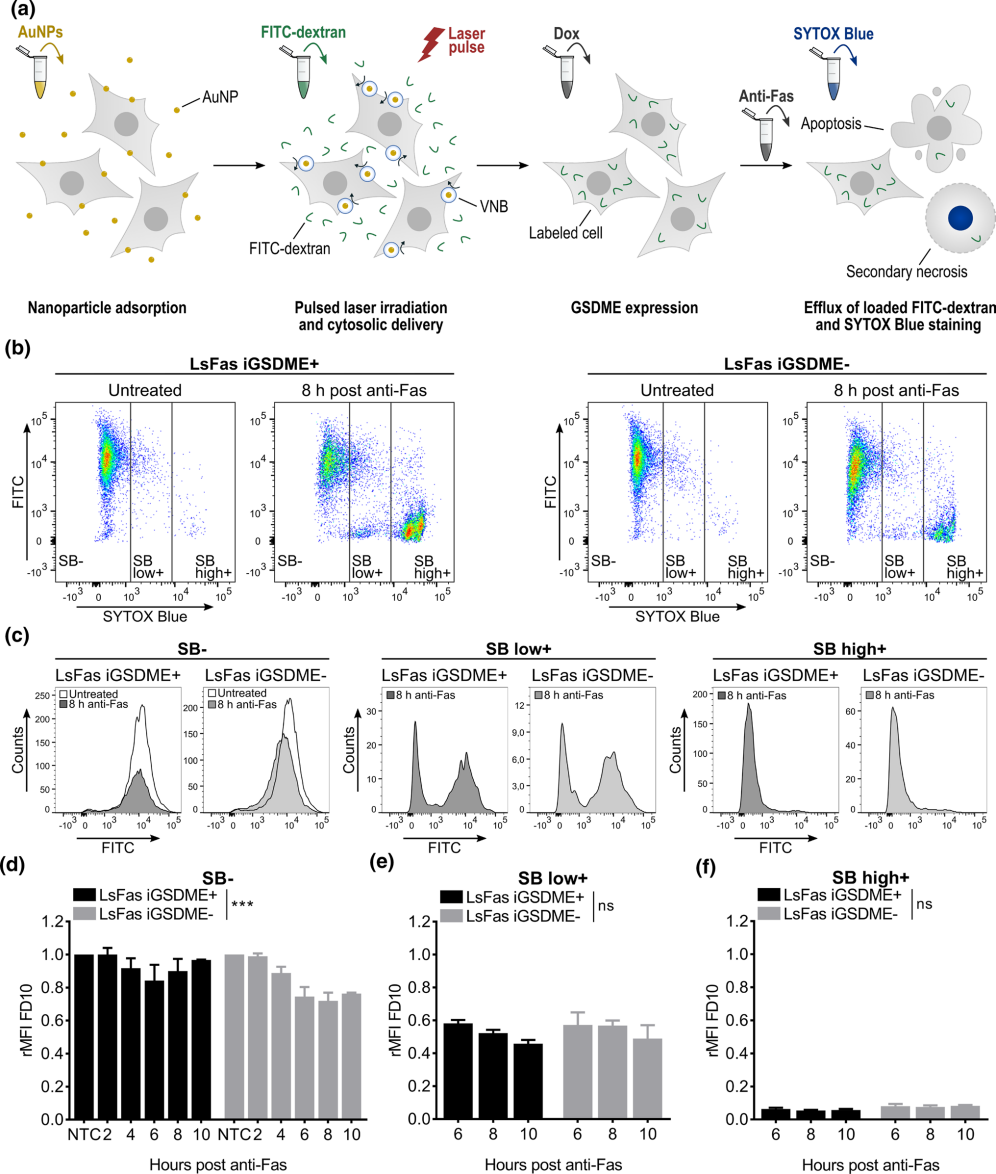
Efflux of FITC-labeled dextrans 10 kDa (FD10) from L929sAhFas iGSDME cells during apoptosis-driven secondary necrosis. **a** Principle of monitoring efflux of FITC-labeled dextrans after photoporation-based dextran loading. **b–f** Flow cytometry analysis of FD10 release in L929sAhFas iGSDME with (L929sAhFas iGSDME+) and without (L929sAhFas iGSDME−) doxycycline-induced GSDME expression when treated with anti-Fas. **b** Scatter plots of L929sAhFas iGSDME in presence (left) and absence (right) of GSDME expression untreated and after 8 h treatment with anti-Fas. **c** Histogram plots representing the distribution of the FD10 signal in the three zones of SB staining: SB− (left), SB low+ (middle) and SB high+ (right). **d** Graph representing the relative mean fluorescence intensity (rMFI) of FD10 in the SB− population (relative to the untreated SB− population) as a function of anti-Fas treatment. **e** Graph representing the relative mean fluorescence intensity (rMFI) of FD10 in the SB low+ population (relative to the untreated SB− population) as a function of anti-Fas treatment. **f** Graph representing the relative mean fluorescence intensity (rMFI) of the SB high+ population (relative to the untreated SB− population) as a function of anti-Fas treatment. *AuNP*, gold nanoparticle; *Dox* doxycycline; *FD* FITC-dextran; *GSDME* gasdermin E; *LsFas* L929sAhFas; *NTC* non-treatment control; *SB* SYTOX Blue; *VNB* vapor nanobubble

Although monitoring the influx of Texas Red-labeled dextrans provided insight into the molecular weight of molecules that can enter during apoptosis-driven secondary necrosis, we aimed to evaluate whether the same conclusions are reached when monitoring the efflux of macromolecules. Monitoring efflux should better reflect the physiological situation where intracellular content like Damage Associated Molecular Patterns (DAMPs) or even cell organelles are released from dying cells. Instead of Texas Red-labeled dextrans, of which we observed that they tend to interact with intracellular constituents, we used FITC-labeled dextrans, which are inert in cells [42]. For delivery of FITC-labeled dextrans in the cytosol of L929sAhFas cells, we used nanoparticle-sensitized photoporation as an emerging intracellular delivery technique that minimally perturbs the cellular homeostasis (Fig. 4a) [23, 24, 27]. L929sAhFas cells were first incubated with cationic AuNPs, which attach to the plasma membrane. After washing away unbound AuNPs, cells were irradiated with a 5 ns laser pulse (*λ* = 532 nm, 0.86 J/cm^2^), resulting in the formation of transient pores in the plasma membrane through which the fluorescently labeled dextrans can diffuse into the cytosol.

First, we optimized the AuNP concentration as a function of delivery efficiency and cell metabolic activity. To maximize cell loading and minimize potential cell cytotoxicity by photoporation, different AuNP concentrations (2, 4, 6, 8 and 16 × 10^7^ AuNPs/mL) were screened using a fixed laser pulse fluence of 0.86 J/cm^2^. We observed an increase in the percentage of cells positive for FITC-labeled dextrans of 10 kDa (FD10) (Fig. S2a), as measured by flow cytometry, and a decrease in metabolic activity, as measured with the CellTiter-Glo® assay (Fig. S2b), for increasing AuNP concentrations. Allowing a 30% reduction in metabolic activity, determined by the ATP content of live cells, the optimal AuNP concentration was set at 6 × 10^7^ AuNPs/ml for all further experiments, in which case near 100% of the cells are FD10 positive. In addition, we tested whether photoporation of FD10 influenced cell death kinetics of L929sAhFas cells when treated with anti-Fas. *Gsdme* WT and KOcl2 L929sAhFas cells were photoporated in the presence of FD10 and cell death kinetics, as determined by SB staining, was compared with untreated control cells. Cell death kinetic measurements of photoporated cells remained unchanged compared to the untreated control cells (Fig. S3). Based on these results, we concluded that photoporation can efficiently deliver FITC-labeled dextrans in L929sAhFas cells without influencing anti-Fas-mediated apoptosis-driven secondary necrosis.

### Efflux of dextrans of 10 kDa occurs independently of gasdermin E expression and cell death kinetics during apoptosis-driven secondary necrosis

Having optimized the cytosolic delivery of FD10 with nanoparticle-sensitized photoporation, we investigated the efflux of the dextrans from L929sAhFas iGSDME cells upon anti-Fas treatment, as a function of the SB signal of the cells (Fig. 4a). Following this strategy, we gated the whole cell population undergoing anti-Fas treatment into: no SB signal (SB-), a low SB signal (SB low+) and a high SB signal (SB high+) (Fig. 4b). Flow cytometry data revealed that both in the presence and absence of GSDME, FD10 was released from the cells when they became positive for the SB-mediated nuclear staining (Fig. 4b, c). Interestingly, a bimodal distribution in the FITC signal was observed in cells with a low SB signal (Fig. 4c, middle panel), which was not observed in the influx experiments. This observation indicates that in the initial stage, when the nucleus of cells gets stained by SB, a part of those cells had already lost FD10 content while the other part was still clearly FD10 positive. In contrast, only a very few SB- cells were negative for FD10 (Fig. 4c, left panel), while cells with a high SB+ signal had practically all lost their dextran content (Fig. 4c, right panel). Of note, these results were observed independently of GSDME expression.

This strong heterogeneity of dextran release between SB− and SB high+ cells is confirmed when plotting the mean fluorescence intensity (MFI) of FD10 in the SB−, SB low+ and SB high+ population, respectively, relative to the untreated SB− population. This normalization is also referred to as the relative mean fluorescence intensity (rMFI) (Fig. 4d–f). Note that we chose to use the rMFI to present the loss of FITC-labeled dextrans, since photoporation delivery efficiency (i.e. the percentage of FD10 positive cells) decreases with increasing dextran size [24, 34]. Furthermore, normalization of the MFI relative to the untreated SB- population allows for easy comparison of the efflux of different sizes of FITC-labeled dextrans as relative differences in dextran delivery after photoporation are taken into account. Only a minimal amount of FD10 content was released from SB− cells (Fig. 4d). Surprisingly, the rMFI decreased slightly but significantly more in the absence of GSDME than in GSDME-reconstituted cells, suggesting that there would be more content release over time in SB− cells when GSDME is lost. This is a counterintuitive result, which is in contrast with our influx data that pointed toward facilitated uptake of dextrans when GSDME pores are formed in SB− cells. However, referring to the prolonged stage of PS-positivity in SB− cells without GSDME expression (Fig. 1h), we hypothesize that the larger drop in rMFI in those cells can be attributed to the prolonged release of FD10-loaded apoptotic membrane blebs in cells lacking GSDME. Taken together, based on these data, we could not claim that GSDME expression facilitates the efflux of small dextrans in SB− L929sAhFas iGSDME cells upon anti-Fas treatment.

In strong contrast to the SB- population, SB high+ cells have lost almost all of their FD10 content irrespective of GSDME expression (Fig. 4f). While the SB low+ population had an intermediate rMFI level, being the result of a FD10-positive and –negative population, there was no difference between GSDME-expressing and non-expressing cells (Fig. 4e). Interestingly, treating cells for longer time periods with anti-Fas resulted in a decreased rMFI of FD10 in the SB low+ population (Fig. 4e). These observations would indicate that slower-dying cells, which enter the SB low+ stage at later time points, have a stronger tendency to release more content early-on in the dying process (SB low+ stage) before attaining a full SB signal, while the opposite would be the case for faster-dying cells, which are stained by SB shortly after anti-Fas treatment. A possible explanation for this would be that slower-dying cells would eventually become more permeabilized during the sublytic phase than faster-dying cells, which progress more rapidly toward the SB low+ stage.

### Efflux of dextrans is size dependent but gasdermin E independent during apoptosis-driven secondary necrosis

**Fig. 5:**
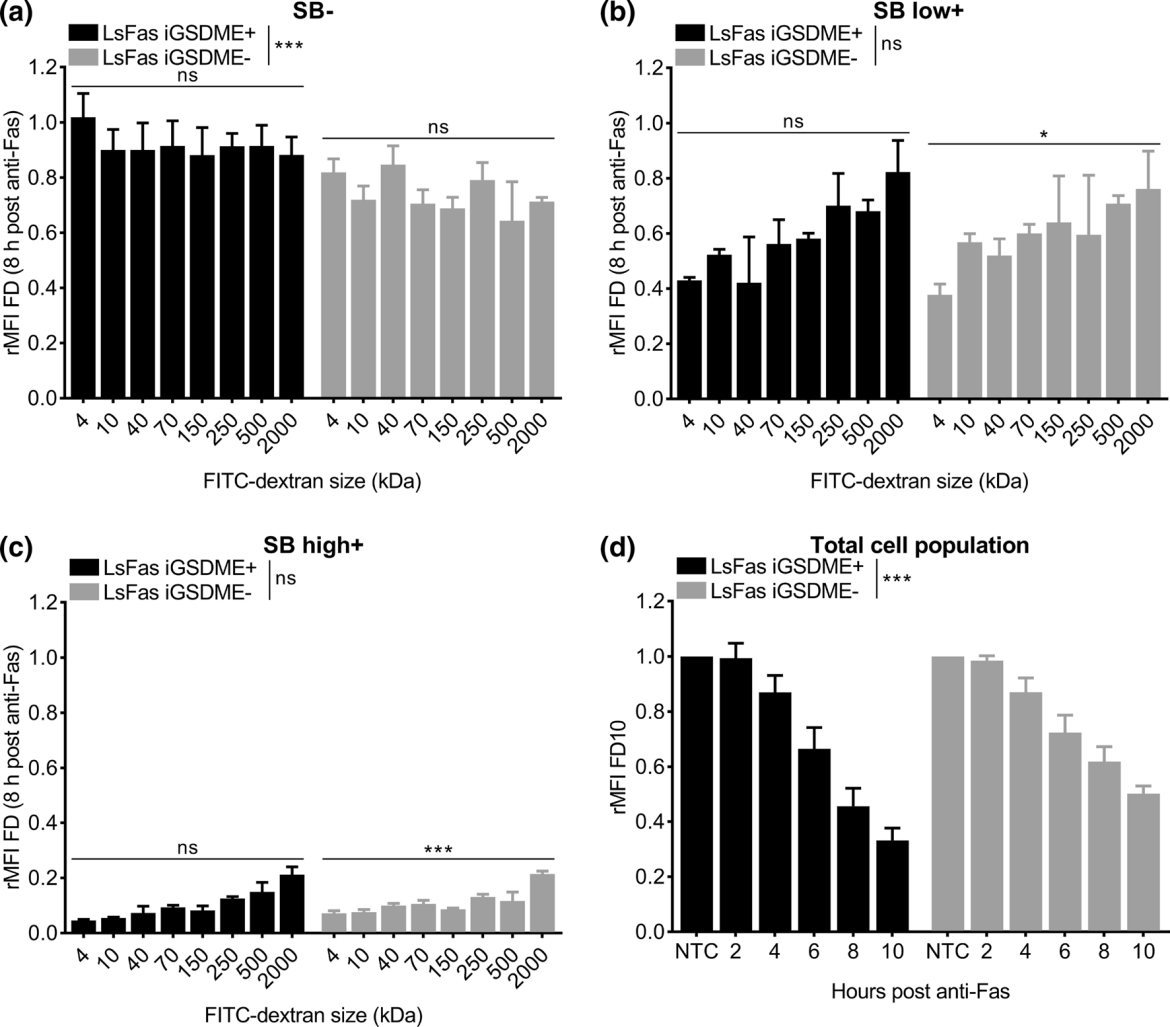
Efflux of FITC-labeled dextrans of different sizes from L929sAhFas iGSDME cells during apoptosis-driven secondary necrosis. **a–d** Flow cytometry analysis of FITC-labeled dextran release in L929sAhFas iGSDME cells with (L929sAhFas iGSDME+) and without (L929sAhFas iGSDME−) doxycycline-induced GSDME expression upon treatment with anti-Fas. **a–c** Graphs representing the relative mean fluorescence intensity (rMFI, relative to the untreated SB− population) for increasing sizes of FITC-labeled dextrans **a** in the SB− population, **b** in the SB low+ population and **c** in the SB high+ population after 8 h treatment with anti-Fas. **d** Graph representing the relative mean fluorescence intensity (rMFI) of the total cell population (relative to the total untreated cell population) as a function of anti-Fas treatment. *FD* FITC-labeled dextran, *LsFas* L929sAhFas, *NTC* non-treatment control

To evaluate whether the release of dextrans from anti-Fas-treated L929sAhFas iGSDME cells is size dependent, we monitored the efflux of FITC-labeled dextrans of increasing molecular weights: 4 kDa (FD4), 40 kDa (FD40), 70 kDa (FD70), 150 kDa (FD150), 250 kDa (FD250), 500 kDa (FD500) and 2000 kDa (FD2000). Efflux in SB- cells was size independent, albeit that L929sAhFas iGSDME- cells had lost more FITC-labeled dextrans compared to L929sAhFas iGSDME+ cells (Fig. 5a). This supports our previous hypothesis that dextran loss is dominated by blebbing in SB- cells, especially in the absence of GSDME. In contrast, SB high+ cells have lost almost all FITC-labeled dextran content of all sizes independently of GSDME expression (Fig. 5c). Nevertheless, a slight size-dependent trend was seen, indicating less dextran release with increasing molecular weight, which was significant in the absence of GSDME expression (Fig. 5c). This size-dependent trend was more obvious in SB low+ cells (Fig. 5b), although again only significant for GSDME-deficient cells. Together these data point toward a size-dependent but GSDME-independent release of dextrans as soon as cells start to become positive for SB. Although no clear size cut-off of the GSDME pore could be determined via this strategy, release of FITC-labeled dextrans, in general, is size dependent during apoptosis-driven secondary necrosis. This can be concluded from the stronger release of smaller-sized dextrans in SB low+ cells compared to larger-sized dextrans, which tend to be released rather at the end of permeabilization (Fig. 5a, b, Fig. S4a, b). The fact that larger-sized dextrans are less easily released as compared to smaller-sized dextrans may indicate the presence of another, GSDME-independent, plasma membrane permeabilization subroutine in SB+ cells that allows the release of FITC-labeled dextrans in a size-dependent way. Of note, as limited efflux was observed in SB− cells, the subroutine promoting efflux of FITC-labeled dextrans coincided with SB staining in our cells. Importantly, as concluded from previous sections, cell death kinetics measured by SB-mediated nuclear staining is delayed in L929sAhFas iGSDME cells in the absence of GSDME expression (Fig. 1f). Therefore, one can expect that a delayed efflux of FITC-labeled dextrans is similar to the delay of influx (not corrected for cell death kinetics) in L929sAhFas iGSDME cells in absence of GSDME expression (Fig. 2c, d). Indeed, when evaluating the rMFI (relative to the total untreated cell population) of the total cell population (without gating for SB signal), overall a slower efflux of FITC-labeled dextrans was observed in L929sAhFas iGSDME− cells as a function of anti-Fas treatment (Fig. 5d). Although efflux of FITC-labeled dextrans through the GSDME pore seems unlikely, this observation highlights the importance of GSDME in the overall cellular release of FITC-labeled dextrans. Altogether, our results suggest that GSDME contributes to a larger set of mechanisms steering membrane permeabilization during apoptosis-driven secondary necrosis and by consequence content release.

## Discussion

Plasma membrane permeabilization following apoptosis-driven secondary necrosis has always been perceived as a non-regulated process of apoptotic cells in the absence of sufficient phagocytic cell capacity [2, 43]. Recently, it was shown that this plasma membrane permeabilization is a regulated process driven by caspase-3-mediated activation of GSDME [12]. This finding indicates that the cell can accelerate the process of permeabilization by engaging GSDME-mediated release of intracellular content, which affects the inflammatory response [44]. Although pore formation by GSDMA3 and GSDMD has already been extensively studied [19, 21, 22], the kinetics and size characteristics of GSDME pore formation during apoptosis-driven secondary necrosis are currently unknown. Determining the degree of membrane permeabilization and identifying molecular sizes able to pass the plasma membrane upon GSDME expression may give insights into the membrane destabilizing behavior of GSDME and its role in progressing apoptotic cells toward secondary necrosis.

In the first part of this study, we showed that GSDME expression contributes to apoptosis-driven secondary necrosis in L929sAhFas cells by accelerating cell death kinetics measured by SB staining. This is consistent with the findings of Rogers et al. [12] stating that GSDME is necessary for the quick progression of apoptotic cells toward secondary necrosis. Additionally, we demonstrated that GSDME expression is dispensable for Fas-induced PS exposure in L929sAhFas, which is an early subroutine of apoptosis. As GSDME expression does accelerate plasma membrane permeabilization, dying cells remain longer in the PS single-positive stage in the absence of GSDME expression. The physiological consequences of how the absence or presence of GSDME determines the lytic phase of the cell death resulting in a prolonged or shortened PS single-positive stage during which cellular content is contained, are currently unknown. It is tempting to speculate that prolonged exposure of eat-me signals on intact cell membranes in the absence of GSDME expression facilitates ‘silent’ clearance of these cells as phagocytes that engulf intact apoptotic cells secrete anti-inflammatory cytokines such as TGFβ and IL-10 while simultaneously suppressing pro-inflammatory cytokines [1, 45–47]. In contrast, DAMP release from perforated necrotic cells attract pro-inflammatory immune cells such as macrophages and neutrophils that release pro-inflammatory cytokines [4, 5, 48]. As such, GSDME expression was shown to increase phagocytosis of tumor cells by macrophages as well as the number and cytolytic activity of tumor-infiltrating natural-killer and CD8+ T lymphocytes, resulting in reduced tumor growth [44].

In the second part of this study, we monitored the influx and efflux of dextran molecules of various sizes in L929sAhFas iGSDME cells with and without GSDME expression. Our results based on the influx of Texas Red-labeled dextrans suggest that GSDME-dependent pore-formation in the sublytic phase, before SB-mediated nuclear staining, allows the passage of molecules with sizes up to 70 kDa, while influx is reduced and delayed in the absence of GSDME expression. This is consistent with earlier reports presenting that GSDMD and GSDME pores in sublytic cells upon pyroptotic stimuli are crucial for the release of cytokines such as active IL-1β (18 kDa) [49–51]. Additionally, our influx-based results imply that GSDME also facilitates the uptake of larger dextrans in SB+ cells, which possibly elucidates the contribution of GSDME to final cell lysis. From our influx experiments, we could estimate that GSDME-driven plasma membrane permeabilization favors the passage of molecules up until ~ 125 kDa. This is consistent with Evavold et al. [49] reporting that macromolecules such as lactate dehydrogenase (144 kDa) were unable to pass GSDMD pores and were only released after complete cell lysis. Although our results based on the influx of dextrans allowed us to conclude that GSDME favors the entrance of macromolecules, a clear cut-off size for molecules able to pass GSDME pores was not observed since we report a decrease in the uptake of Texas Red-labeled dextrans with increasing sizes. These observations might suggest that at any moment during cell death, permeabilization of plasma membranes may involve pores of different sizes that are simultaneously present in the cell population referring to the presence of alternative pore-forming molecules or less controlled pore-formation by GSDME. However, the formation of GSDME membrane pores of different sizes only seems plausible in the case of a plasma membrane-destabilizing mechanism such as the carpet-like model or the formation of toroid-like pores since oligomerization and formation of discrete β-barrel-shaped pores are dependent on thermodynamic stability. Nevertheless, the presence of different pore sizes could indicate that intermediate pores are formed that undergo a growing process until they reach their final stable form as shown for GSDMD pores [21, 22].

While influx experiments provided us with valuable insights regarding membrane permeabilization during apoptosis-driven secondary necrosis, we were keen to investigate the effect of this process on the efflux of FITC-labeled dextrans. Monitoring efflux should better reflect the physiological situation where intracellular content is released from dying cells. Interestingly, more intermediate dextran sizes are available with the FITC fluorophore, which could aid in drawing more precise conclusions about GSDME. We used nanoparticle-sensitized photoporation as an efficient intracellular delivery technique, of which we could show that it does not interfere with apoptosis kinetics. However, upon triggering of apoptosis-driven secondary necrosis, we did not find a contribution of GSDME to the efflux of FITC-labeled dextrans from L929sAhFas iGSDME cells. In addition, SB high+ cells released almost all FITC-labeled dextrans while in our influx experiments, TR2000 failed to enter in most of the SB+ cells. These observations suggest that other, GSDME-independent, subroutines exist that allow the release of FITC-labeled dextrans. The existence of different subroutines supporting membrane permeabilization during cell death has recently been shown by Kayagaki et al. They report that the cell-surface protein nerve injury-induced protein 1 (NINJ1) contributes to plasma membrane permeabilization during apoptosis-driven secondary necrosis, pyroptosis and necroptosis next to GSDME, GSDMD and mixed lineage kinase domain-like (MLKL) [52]. Which subroutine is responsible for the efflux of FITC-labeled dextrans in our system remains elusive, but similar to the influx of Texas Red-labeled dextrans, efflux of FITC-labeled dextrans occurs in a size-dependent manner. As to why GSDME pores seem to exclude FITC-labeled dextrans, we cannot rule out an electrostatic effect. FITC-labeled dextrans are anionic while Texas Red-labeled dextrans are more neutral. Recently, the GSDMD pore was shown to be predominantly negatively charged preventing the passage of negatively charged cargos [20].

## Conclusions

We developed two strategies to elucidate the influence of GSDME in apoptosis-driven secondary necrosis and gained insights into the pore-forming and membrane permeabilizing behavior during this process. While a size dependency was observed for both influx and efflux of fluorescently labeled dextrans, we could only attribute an altered influx pattern to GSDME presence. Altogether, our results point to the existence of different subroutines that simultaneously regulate the passage of compounds during the cellular permeabilization process during apoptosis-driven secondary necrosis.

## Supplementary Information

Below is the link to the electronic supplementary material.

Supplementary file1 (PDF 938 kb)

## Data Availability

The datasets generated during and/or analyzed during the current study are available from the corresponding author on reasonable request.
