# Supplementary material for: Plasma membrane perforation by GSDME during apoptosis-driven secondary necrosis
Source: Cell Mol Life Sci. 2021 Dec 31;79(1):19. doi: 10.1007/s00018-021-04078-0 (PMC8720079; doi:10.1007/s00018-021-04078-0)
Supplement: Supplementary file 1 — Supplementary file1 (PDF 938 kb) [file 18_2021_4078_MOESM1_ESM.pdf]

SUPPLEMENTARY FILE SUPPORTING:

Title: Plasma membrane perforation by GSDME during apoptosis-driven secondary necrosis.

In Cellular and Molecular Life Sciences

Author information

Authors: Elke De Schutter [1,2,3,†], Jana Ramon [4,†], Benjamin Pfeuty [5], Caroline De Tender [6,7], Stephan Stremersch [4], Koen Raemdonck [4], Ken Op de Beeck [3,8], Wim Declercq [1,2], Franck B. Riquet [1,2,9,§] and Kevin Braeckmans [4,§] and Peter Vandenabeele [1,2,§,*]

[†] These authors contributed equally to this work

[§] Shared senior authorship

[1] VIB Center for Inflammation Research, 9052 Ghent, Belgium

[2] Department of Biomedical Molecular Biology, Ghent University, 9052 Ghent, Belgium

[3] Center of Medical Genetics, University of Antwerp and Antwerp University Hospital, 2650 Antwerp, Belgium.

[4] Laboratory of General Biochemistry and Physical Pharmacy, Faculty of Pharmaceutical Sciences, Ghent University, 9000 Ghent, Belgium

[5] Université de Lille, CNRS, UMR 8523 - PhLAM - Physique des Lasers Atomes et Molécules, F-59000 Lille, France

[6] Department of Applied Mathematics, Computer Science and Statistics, Ghent University, 9000 Ghent, Belgium

[7] Plant Sciences Unit, Flanders Research Institute for Agriculture, Fisheries and Food, 9820 Merelbeke, Belgium

[8] Center for Oncological Research, University of Antwerp and Antwerp University Hospital, 2610 Antwerp, Belgium

[9] Université de Lille, 59000 Lille, France

*Correspondence: Peter.Vandenabeele@irc.vib-ugent.be (P. Vandenabeele)

Table S1 sgRNA sequences, PCR and sequencing primers used for *Gsdme* CRISPR-Cas9 gene editing

Sequence	Forward Sequence (5'→3')	Reverse complement (5'→3')
Guide sequence	TCCCAATAGCCCCGCTCTTA	TAAGAGCGGGGCTATTGGGA
Primers	GCATTCAATACATGGTTTTTGG	TAATCACCCCTAGGCTCTGG

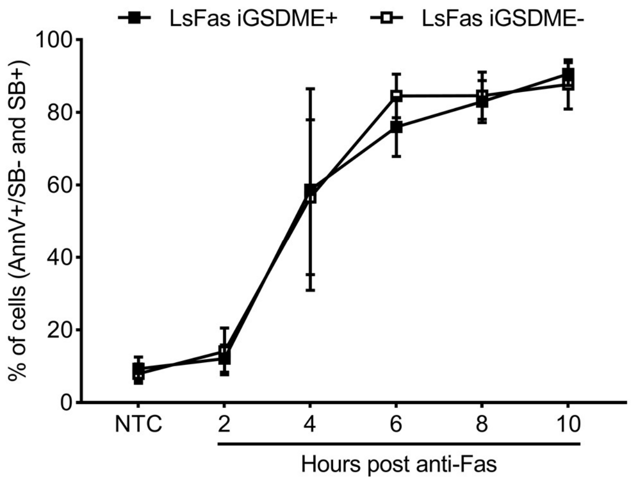

Fig. S1 Total amount of cell death, represented by the sum of the AnnV+/SB- and SB+ cells, in L929sAhFas cells with (L929sAhFas iGSDME+) or without (L929sAhFas iGSDME-) doxycycline-induced GSDME expression when treated with anti-Fas. AnnV, Annexin V; LsFas, L929sAhFas; NTC, non-treatment control; SB, SYTOX Blue

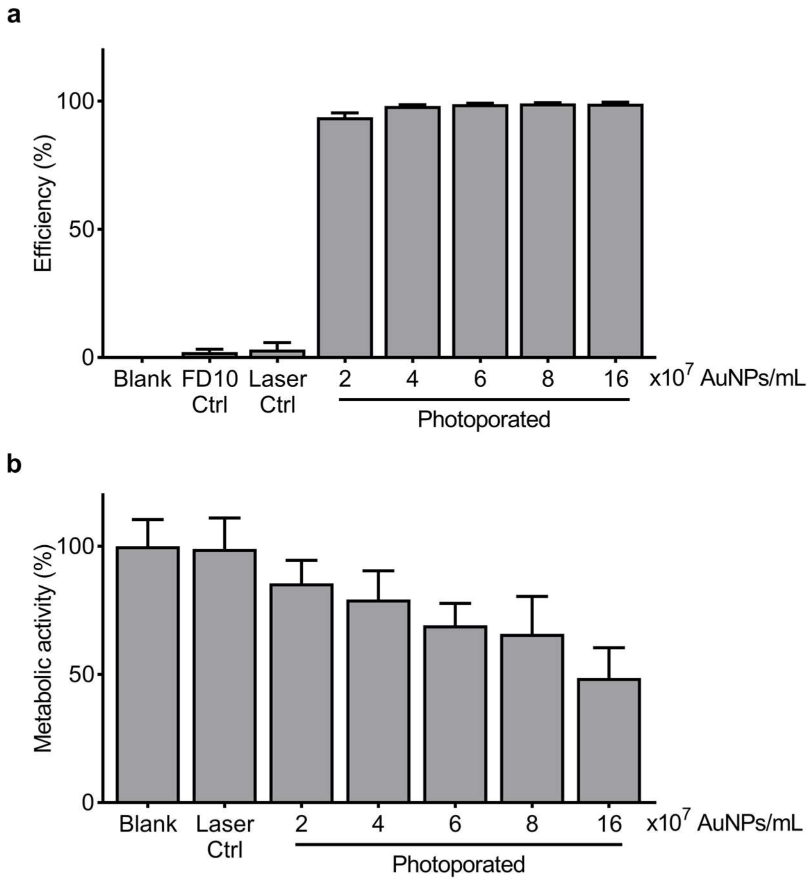

Fig. S2 Optimization of AuNP concentrations using a fixed laser fluence of 1.6 J/cm² in L929sAhFas cells. **a** Delivery efficiency of FITC-labeled dextran of 10 kDa (FD10) in function of increasing AuNP concentrations. **b** Metabolic activity in function of increasing AuNP concentrations. AuNPs, gold nanoparticles; Ctrl, control

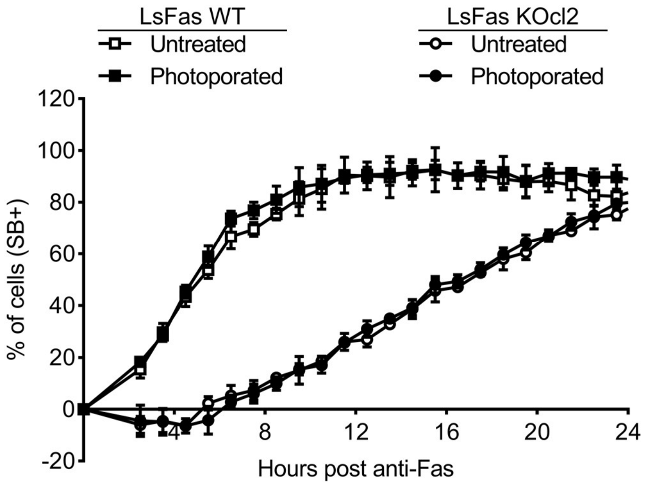

Fig. S3 Cell death kinetics, as determined by the SB staining, in untreated and photoporated cells, both in *Gsdme* WT and *Gsdme* KOcl2 L929sAhFas cells in function of anti-Fas treatment. KO, knockout; LsFas, L929sAhFas; SB, SYTOX Blue; WT, wild-type

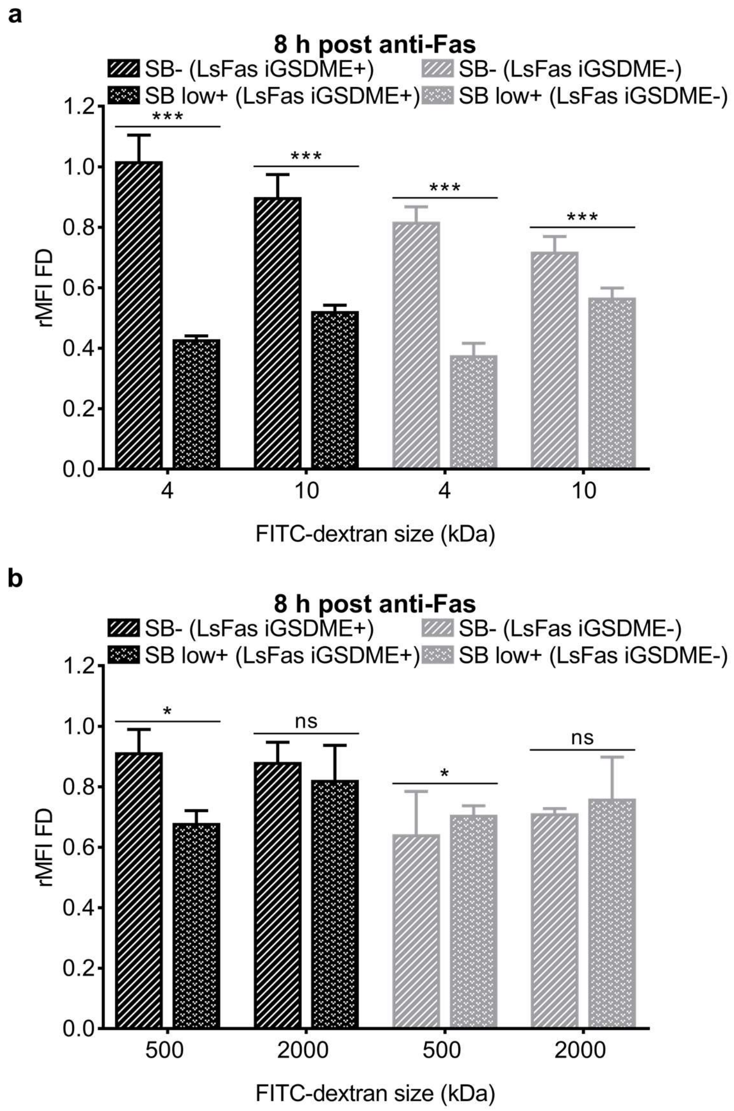

Fig. S4 Comparison of the relative mean fluorescence intensity (rMFI, relative to the untreated SB- population) for different sizes of FITC-labeled dextrans, between the SB- and SB low+ population after 8 h of anti-Fas treatment. **a** FITC-labeled dextran 4 kDa (FD4) and 10 kDa (FD10). **b** FITC-labeled dextran 500 kDa (FD500) and 2000 kDa (FD2000). FD, FITC-labeled dextran; LsFas, L929sAhFas; SB, SYTOX Blue
